# Cinnamon Essential Oil Delivery Systems: Preparation Processes, Packaging Forms, and Industrialization Potentials

**DOI:** 10.1002/fsn3.70814

**Published:** 2025-08-29

**Authors:** Liquan Zhou, Yahong Cai, Yanjing Li, Yimiao Zhou, Zuowei Xiao

**Affiliations:** ^1^ The First Affiliated Hospital of Hunan University of Chinese Medicine Hunan University of Chinese Medicine Changsha China; ^2^ Hunan Engineering and Technology Research Center for Health Products and Life Science, School of Pharmacy Hunan University of Chinese Medicine Changsha China; ^3^ Homologous Innovation Laboratory of Medicine and Food Hunan University of Chinese Medicine Changsha China; ^4^ School of Traditional Chinese Medicine Changsha Medical University Changsha China; ^5^ Xiangyin Campus, Xiangxing College Hunan University of Chinese Medicine Yueyang China

**Keywords:** active packaging, cinnamon essential oil, delivery system, industrial production, intelligent packaging

## Abstract

Cinnamon essential oil (CEO), recognized for its broad‐spectrum antimicrobial and antioxidant properties, is a natural alternative to synthetic preservatives. However, its high volatility, low water solubility, and strong odor limit direct application. This review examines advanced delivery systems—including emulsions, nanocarriers, molecular inclusion complexes, microcapsules, and liposomes—designed to enhance CEO stability, mask undesirable flavors, and enable controlled release. These systems are integrated into active, edible, and intelligent packaging forms. Active packaging extends food shelf life by inhibiting microbial growth, while intelligent packaging incorporates anthocyanin indicators for real‐time freshness monitoring via pH‐responsive color changes. Despite promising laboratory results, industrialization faces challenges such as high equipment costs, expensive raw materials, and stability issues. Future advancements require process optimization, low‐cost carrier development, and composite material strategies to promote functional, intelligent, and scalable CEO delivery systems.

## Graphical abstract

This review systematically evaluates delivery systems (nanoemulsions, microcapsules, and Pickering emulsions) for encapsulating CEO, comparing their preparation processes, controlled‐release efficiency, and scalability. By integrating material innovation, intelligent packaging design, and sustainable strategies, the study provides actionable insights to advance CEO‐based technologies toward functional, eco‐friendly, and industrially viable food preservation solutions.

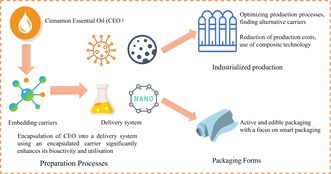

Food packaging is a key link in the food industry chain to ensure quality, extend shelf life, and convey information. In the complex circulation process of food from production to consumption, packaging materials act as physical barriers, effectively slowing down food oxidation, mold, and corruption, and maintaining its nutrients and sensory qualities by blocking the intrusion of oxygen, moisture, and microorganisms (Yang et al. 2025). Traditional packaging technology is mainly passive protection, focusing on isolation of physical, chemical, and microbial contamination, but it is difficult to meet the diversified needs of consumers for food freshness, safety, and environmental protection (Culas et al. 2024). In this context, active packaging has emerged, through the integration of antimicrobial agents, antioxidants, and other functional substances into the packaging system, to actively regulate the microenvironment of the food, to delay spoilage and improve safety, and has become an important direction of innovation in the field of food packaging (Ribeiro‐Santos et al. 2017).

According to the World Health Organization (WHO), the annual global food loss due to microbial contamination is serious, and the controversy over the safety of synthetic preservatives (e.g., benzoic acid, potassium sorbate) is becoming increasingly prominent (Piper and Piper 2017). In recent years, cinnamon essential oil (CEO) has shown promising applications in the field of active food packaging due to its excellent antimicrobial (e.g., inhibition of *Escherichia coli* , *Staphylococcus aureus* ) and antioxidant activities (e.g., scavenging of free radicals, reduction of lipid oxidation). CEO is a naturally occurring volatile mixture derived from the bark of the *Cinnamomum* tree, and its main components are cinnamaldehyde (≥ 65%), cinnamyl acetate, eugenol, etc. (Borges et al. 2024). As a Generally Recognized as Safe (GRAS) material approved by the US FDA, CEO can play a role in disrupting microbial cell membranes, inhibiting enzyme activity, and other mechanisms, which is in line with consumer demand for natural and healthy food.

Studies have shown that CEO is effective in extending the shelf life of perishable foods such as fruits, vegetables, meat, and dairy products, while improving their organoleptic qualities such as color and odor (Mao et al. 2024). However, the high volatility, low water solubility, and strong pungent odor of CEO make it a double challenge for direct application. On the one hand, essential oils are easily lost through volatilization, resulting in a decrease in antimicrobial efficiency; on the other hand, high localized concentrations may penetrate into food matrices, especially in porous or odor‐absorbent food materials, thus altering the original flavor profile of the food (e.g., masking the original aroma of meats, affecting the sweet–sour ratio of fruits and vegetables; Marsin et al. 2020). For example, when baked goods such as bread are preserved in packaging materials containing CEO, the bread may take on a distinct cinnamon flavor if not properly controlled (Balaguer et al. 2013). In addition, excessive migration of cinnamaldehyde in dairy products may introduce a spicy flavor (Ali et al. 2021).

To solve the above problems, research in recent years has focused on encapsulating CEOs through delivery systems so as to achieve targeted protection and controlled release of CEOs (Luo et al. 2024). Among them, microencapsulation technology can effectively control the release rate and amount of CEO by encapsulating CEO in the wall material of microcapsules, thus reducing the interference of its odor on food flavor to a certain extent (Huang et al. 2022). In addition, delivery systems such as nanoemulsions (NEs) also reduce the volatility of CEO and the escape of odor by modifying the composition and structure of the system (Liang et al. 2022). By optimizing the type and amount of surfactant and controlling the droplet size of the emulsion, the stability of CEO in the packaging system can be effectively improved, which in turn reduces its impact on food flavor due to volatilization (Zhang, Ezati, et al. 2023). Chuesiang et al. (2021) investigated the antimicrobial activity of CEO NEs, bulk CEO, and sodium hypochlorite, which are the three types of CEO, on refrigerated Asian sea bass fillets. Compared with untreated samples, 11,429 mg/L of CEO NE reduced the initial bacterial counts by about 0.5–1.5 log CFU/g. The NE successfully inhibited the growth of the above‐mentioned bacteria during the storage period of the fish fillets (4°C ± 2°C), especially against *Vibrio parahaemolyticus* , and its antimicrobial effect was superior to that of regular CEO and sodium hypochlorite. Chen, Deng, et al. (2024) developed a bioactive sponge pad consisting of oxidized bacterial cellulose (OBC) and CEO‐loaded microcapsules (CGCM), the latter of which was prepared by complex coagulation of chitosan and gum arabic. The dosage of CEO was 0.2 mL. Experiments demonstrated that electrostatic interactions and hydrogen bonding between CGCM and the OBC sponge pad matrix enhanced the thermal stability, water absorbency, and tensile strength of the sponge pad. In addition, the material exhibited high antioxidant and antimicrobial properties, especially in the gas phase, with an antimicrobial rate of up to 65%. When the prepared sponge mat was applied to meat preservation, the freshness of meat could be maintained at 4°C, extending the shelf life from 4 days to about 10 days.

Although these technologies have made significant progress in the laboratory stage, their industrialized application still faces bottlenecks such as high equipment costs and poor stability. This review systematically explores the core elements of the CEO delivery system, including carrier material design, preparation process optimization, packaging form innovation, and industrial production path. By comparing the feasibility of industrialization of the CEO delivery system and its advantages and disadvantages, this review balances its bioactivity retention, controlled release, and flavor compatibility and provides a theoretical basis for the functionalization, intelligence, and large‐scale application of the CEO delivery system.

## 1 Classification of Carrier Materials

The use of carrier materials to encapsulate CEO into delivery systems can significantly enhance its bioactivity and utilization. Ideal carrier materials should have good biocompatibility, stability, a high loading rate, and controlled release ability. Carrier materials can be categorized into three main types: natural polymers, synthetic polymers, and composite polymers, with significant differences in their performance characteristics and applications.

Natural polymeric materials can balance the performance and cost of preparing delivery systems, such as polysaccharides (starch, cellulose, chitosan, sodium alginate, etc.) and proteins (gelatin, gum arabic, whey protein, etc.; KV et al. 2024; Zhang, Wang, et al. 2024; Zhang, Pu, et al. 2024). Most of them are from a wide range of sources, biocompatible, and degradable, which meets the environmental needs of modern food packaging. However, they have a medium encapsulation effect and moderate slow‐release performance and are mainly used in direct food contact scenarios, such as fruit and vegetable coating and edible packaging (Qiu et al. 2022). At the same time, they are susceptible to environmental humidity and pH and have poor stability, such as chitosan film that is prone to moisture absorption and swelling under high humidity, leading to rapid release of CEO; gelatin film that is prone to tearing; and starch film that has low water resistance. Hashim et al. (2024) utilized biopolymer‐stabilized microcapsules to enhance the physicochemical stability and antimicrobial properties of CEO. Experiments were conducted to prepare microcapsules by freeze‐drying using berry wax (RW) as a solid lipid carrier, compounded with whey protein (WYN), maltodextrin (MDN), and gum arabic (GMC), respectively, as wall materials. Experiments showed that the combination of WYN and RW as wall materials had the highest encapsulation efficiency (93.4%) for chicken breast preservation. The WYN/RW/CEO microcapsules had a uniform particle size (200–300 nm), the most stable CEO release, the strongest antioxidant capacity, and the largest inhibitory circle against *E. coli* and *S. aureus* (40 mm).

Starch can be modified, such as through pasteurization, esterification, etherification, and other treatments, to improve its solubility and film‐forming properties (Mokhtari et al. 2024). For example, starch can be prepared into starch‐based materials such as maltodextrin by enzymatic low hydrolysis, refining, and spray drying. Cyclodextrins, a series of cyclic oligosaccharides produced by the action of cyclodextrin glucosyltransferase on starch, also belong to starch‐based materials. Gao et al. (2023) prepared starch– cinnamaldehyde complexes by wet (aqueous ethanol solution) and dry (solvent‐free) methods at different temperatures. The wet encapsulation rate (15.25–62.38 mg/g) was significantly higher than the dry method (12.39–17.20 mg/g), where 50% (w/w) ethanol concentration optimized the complexing efficiency. The complex prepared with 50% ethanol at 75°C had the highest content of cinnamaldehyde (62.38 mg/g), which could be released continuously for more than 2 weeks at 50°C and 57% humidity, and significantly suppressed *S. aureus* and *E. coli* . Gelatin, with advantages such as good film‐forming and edible properties, is widely used in functional films, food preservation, and active packaging. Akbari et al. (2024) explored the effects of bacterial cellulose nanocrystals—fish gelatin/CEO nano‐coatings containing different concentrations of CEO (1.2, 1.8 and 2.4 mL/L)—on the ‘Red Gold’ nectarine fruits during cold storage (4°C, 60 days). It was shown that the coating treatment delayed fruit ripening, and increasing the essential oil concentration effectively enhanced the coating effect.

Chitosan is a functional polysaccharide derivative of natural chitin modified by partial deacetylation, with excellent film‐forming and broad‐spectrum antimicrobial activities (Herrera‐González et al. 2024). The abundant amino groups in its molecular chain can be protonated in an acidic medium to form cationic polyelectrolytes, which can build stable complexes with negatively charged CEO molecules through electrostatic complexation. At the same time, its film‐forming properties can physically block the volatilization of essential oils and reduce the diffusion of odors into the food matrix (Thi Hang Phuong et al. 2024). Dissolve chitosan in dilute acid solution, then add CEO and mix well to form a coating solution. The coating solution is applied to the surface of fruits and vegetables by dipping, spraying, or brushing to form a protective film. The protective film can not only block the entry of external oxygen, reduce the respiration rate, and delay the aging of fruits and vegetables, but also realize the controlled release of CEO and inhibit microbial contamination. For example, Singla et al. (2022) prepared a composite formulation of 1%, 2%, and 3% chitosan with 0.25% and 0.50% cinnamon oil, respectively, to treat pomegranate seeds by the dip‐coating method. The results showed that all the treatments prolonged shelf life, improved postharvest quality, and reduced microbial population during 15 days of storage. The best results were obtained with 2% chitosan +0.25% cinnamon oil, which increased the total phenolic content and antioxidant activity by 18% and 16%, respectively, and reduced the microbial activity by 45% compared with the control group at the end of the storage period.

Synthetic polymers such as polylactic acid (PLA), polyvinyl alcohol (PVA), and polybutylene adipate (PBAT) have excellent mechanical properties, high slow‐release properties, and are suitable for high‐strength packaging. Examples include meat vacuum packaging bags and liquid food containers. However, their embedding effect is low, and chemical synthetic raw materials may bring security controversy and long degradation cycles; the application of environmental factors needs to be considered (Parin 2023). PLA has excellent thermal stability, mechanical properties, and processability and can be made into packaging containers of various shapes, releasing CEO during slow degradation (de Castro et al. 2024). PVA's water solubility is good, it is easy to form a film, and it has a strong affinity for the CEO; made of the film packaging can moderately dissolve in contact with food moisture and can regulate the humidity inside the package to reduce water loss when used for preservation of fruits and vegetables (Taktak et al. 2024). Sharma et al. (2023) developed a PLA/PBAT composite film containing titanium dioxide and CEO and systematically evaluated its physicochemical properties and antimicrobial function. It was shown that the film with 7% CEO exhibited the best UV barrier, hydrophobicity, and antimicrobial activity and significantly reduced weight loss when applied to cheese packaging.

Composite polymer material refers to two or more different properties of polymer materials, through physical or chemical methods of composite materials, which usually have more excellent packaging performance than a single polymer material. Composite polymer materials have high encapsulation efficiency. Good slow‐release performance and balanced mechanical properties make them suitable for multifunctional packaging (e.g., smart indicators, high barrier coatings), but the preparation process is complicated and costly, limiting large‐scale applications (Guo et al. 2024). Tu et al. (2023) prepared CEO‐liposomes (CEO‐Lip), chitosan (CH)‐modified CEO‐Lip (CH‐CEO‐Lip), and CEO modified by sodium alginate (SA) and CH together. Lip (SA‐CH‐CEO‐Lip), and their preservation effects on fresh pork were investigated. The average particle size of SA‐CH‐CEO‐Lip was 178.73 nm, the polydispersity index (PDI) was 0.380, and the zeta potential was −23.83 mV. The encapsulation efficiency of SA‐CH‐CEO‐Lip reached 89.5%, indicating that it was prepared with high efficiency. The 72‐h in vitro cumulative release rate of SA‐CH‐CEO‐Lip was 71.42%, which was better than that of CEO‐Lip (81.24%). Compared with the CEO‐Lip group, SA‐CH‐CEO‐Lip could effectively inhibit the elevation of pork pH, total volatile nitrogen, and thiobarbituric acid reactants.

## 2 Delivery System

### 2.1 Emulsions

An emulsion is a colloidal system formed by dispersing a liquid in the form of tiny droplets in another immiscible liquid, usually consisting of an oil phase, an aqueous phase, and an emulsifier (Huang et al. 2021). According to the difference between the dispersed phase and the continuous phase, they can be classified as water‐in‐oil (W/O), oil‐in‐water (O/W), and multiple emulsions (e.g., O/W/O). Based on droplet size classification, emulsions can be divided into coarse/macro emulsions (100 nm–200 μm), nano emulsions (20–100 nm), and micro emulsions (5–50 nm) (Muhoza et al. 2023). Droplet size has a significant effect on the physicochemical properties of emulsions; e.g., microemulsions are thermodynamically stable, whereas NEs, coarse emulsions, and multiple emulsions are unstable and prone to gravitational segregation, flocculation, and agglomeration (Zhu et al. 2024). Emulsion delivery systems have the advantages of easy preparation and the ability to modulate CEO release under different conditions, and their structures are easy to incorporate into food systems. However, emulsions are pH and temperature sensitive, have a short storage half‐life, and require protection from light and antioxidant treatment (Shao et al. 2023).

Proteins and surfactants are commonly used in emulsion preparation based on functional properties. Techniques such as homogenization, sonication, high‐pressure homogenization (HPM), and phase transition can be used to prepare emulsions with different droplet size distributions. For example, ultrasound‐assisted emulsification utilizes the cavitation effect of ultrasound to break down droplets and form nanoscale emulsions (Phyo et al. 2025). The ultrasonic method is suitable for laboratory production and mechanically breaks the droplets by the cavitation effect, which is easy to operate and low cost, but difficult to scale up. However, it should be noted that the elevated temperature during ultrasonication may lead to the degradation of cinnamaldehyde. HPM is a method that breaks up droplets to form uniform nanoemulsions (NEs) by forcing the liquid through a narrow gap at high pressure, which is suitable for large‐scale production but with high energy consumption (Khalid et al. 2024). The phase transition temperature (PIT) method is easy to operate, simply heating a mixture of surfactant, oil, and water to near the phase transition temperature, then quenching and cooling with stirring. PIT is suitable for encapsulating thermosensitive essential oils, inducing a phase transition by means of a change in temperature or composition, with less destruction of the active ingredient (Vinh et al. 2020). However, there are complex synergistic interactions between the oil, surfactant, water type, flow rate, and temperature during preparation, and high‐temperature treatment may accelerate the oxidative degradation of the CEO, which can result in potentially less stable emulsions (Chuesiang et al. 2019). The spontaneous emulsification method forms emulsions spontaneously through the interfacial tension between the oil phase, the water phase, and the emulsifier, which is a mild process but has a wide droplet size distribution (Raj et al. 2024).

#### 2.1.1 Microemulsions

Microemulsions are thermodynamically stable colloidal systems formed from oil and water phases, surfactants, and co‐surfactants with a transparent/translucent appearance and low interfacial tension (Al‐Adham et al. 2022). In CEO packaging, microemulsions are often constructed using low‐energy preparation methods such as self‐emulsification and the phase inversion temperature method (PIT). Stable O/W microemulsions can be formed by modulating the type of surfactant (e.g., Tween 80, Span 20), the composition of the oil phase, and the oil‐to‐water ratio.

Microemulsion increases the contact area between CEO and microorganisms through nanoscale dispersion, accelerates the destruction of the cell membrane and the inhibition of enzyme activity, and improves the efficacy of CEO (Shen et al. 2025). The solubilizing effect of surfactants improves the water solubility of CEO and increases its effective concentration in the aqueous phase, while the thermodynamic stability of microemulsions inhibits the volatilization of CEO. In addition, the interfacial membrane structure of the microemulsion can regulate the release rate of CEO e.g., under high ionic strength (0.5 M NaCl), the dissociation of the surfactant layer triggers the rapid release of CEO, which is suitable for immediate antimicrobial scenarios (Wang et al. 2023). The stability of microemulsions is closely related to their thermodynamic properties, and the formation process depends on the hydrophilic–hydrophobic balance (HLB value) of the surfactant molecules and the elasticity of the interfacial film (Ioan et al. 2023). The surfactant molecules form a dense adsorbent layer at the oil–water interface and inhibit the volatilization and oxidation of the CEO. For example, when using Tween 80 as a surfactant, by optimizing the HLB value to 10–12, transparent microemulsions can be formed spontaneously at room temperature with a uniform droplet size distribution and no phase separation during long‐term storage (Kopanichuk et al. 2018). In addition, the salt resistance and pH stability of the microemulsions can be further enhanced by combining surfactants (e.g., Tween 80 with Span 20) or adding co‐surfactants (e.g., ethanol) (Mahmoudzadeh et al. 2022). In food‐active packaging applications, microemulsion‐loaded CEOs are often functionalized by incorporating biopolymer films or edible coatings. For example, Shi et al. (2022) prepared a CEO microemulsion using Tween 80 as a surfactant and anhydrous ethanol as a co‐surfactant. The microemulsion had a K‐m value of 3:1, a preparation temperature of 40°C, an aqueous phase pH of 5, and an O/W type. The microemulsion was highly stable, and the droplet size distribution was concentrated at 81.5 nm, with excellent encapsulation and slow‐release effects. The active package prepared using this microemulsion prolonged the duration and effectiveness of CEO, with 100% repellency against rice elephants within 48 h. González‐González et al. (2025) evaluated the activity of the CEO microemulsion against *E. coli* O157:H7. The emulsion was prepared by mixing 10% CEO (v/v) with 3% Tween 20 or 80 (v/v) and subjecting the mixture to 15 min of ultrasonic treatment. Experiments showed that the minimum inhibitory concentration (MIC) of CEO emulsion with Tween 80 added was 0.85 mg/mL, and the minimum bactericidal concentration (MBC) was 0.95 mg/mL.

#### 2.1.2 NEs

NEs are thermodynamically unstable but kinetically stable oil–water dispersion systems. Surfactant molecules are oriented and aligned at the oil–water interface to form an interfacial film, which stabilizes the dispersion of essential oils in the form of fine droplets in the continuous phase by lowering the interfacial tension and providing spatial site resistance or electrostatic repulsion (Jitpasutham et al. 2024). Depending on the nature of the dispersed and continuous phases, NEs can be classified as O/W or water‐in‐oil (W/O) types, and their stabilization mechanism depends on the interfacial adsorption of surfactants. The small droplet size of NEs can effectively inhibit flocculation and sedimentation during storage, and their transparent or translucent appearance is conducive to enhancing the organoleptic acceptance of food packaging (Ma et al. 2024). Compared with microemulsions, NEs have smaller droplet sizes and narrower distributions (Sahu et al. 2017).

The nanoscale droplet size of NEs inhibits droplet aggregation through electrostatic repulsion or steric hindrance, thereby increasing the contact area between CEO and microorganisms (Sharma et al. 2024). In CEO encapsulation, NEs are commonly constructed using nonionic surfactants such as Tween 80 or Span 20, or biopolymers such as sodium alginate or chitosan as emulsifiers. The solubilization effect of surfactants enhances the effective concentration of CEO in the aqueous phase. By optimizing emulsifier concentration (typically 5%–15%) and oil‐to‐water ratio (1:5–1:10), uniform‐sized O/W type NEs can be obtained (Bahrami et al. 2025; Lakyat et al. 2024).

The stabilization mechanism of NEs is closely related to their interfacial properties. Their stability is significantly affected by factors such as temperature, pH, ionic strength, etc. Emulsification may occur in complex food packaging environments (Elsewedy et al. 2024). Elevated temperatures may cause denaturation of protein emulsifiers and droplet aggregation, while high salt environments may compress the double electric layer and thus reduce electrostatic repulsion (Zhou et al. 2023). The interfacial membrane structure of NEs can regulate the release rate of CEO, and the elasticity and viscosity of the interfacial membrane can be optimized through the combination of emulsifiers (Mahapatra et al. 2024). Therefore, optimization of the oil–water ratio, enhancement of the interfacial film strength, and application of advanced embedding techniques can enhance the stability of the NEs. Liang et al. (2023) carried out a study on the preparation of CEO NEs and screening of emulsifiers. The experiments used an ultrasound‐assisted technique to compare the emulsification effects of sodium caseinate (SC), whey glycoprotein (WGP), soybean isolate protein (SPI), and Tween 80. It was shown that SC (2% concentration) formed the best nanoemulsion with a droplet size of ~180 nm, a zeta potential of −38 mV, a significantly better long‐term storage (30 days) stability than the other emulsifiers, and the highest efficiency of releasing the CEO (78%) in in vitro digestion. Tween 80 had the smallest particle size (120 nm) but was less stable.

In food‐active packaging applications, CEO‐NEs are often functionalized by incorporating biopolymer films/coatings. Applying NEs as coatings on the surface of food packaging can effectively block the penetration of oxygen, moisture, and harmful substances, thus protecting the food from the external environment (Nirmal et al. 2023). On the one hand, the use of nanoemulsion increases the contact area between CEO and the food surface, which improves the efficiency of antibacterial and antioxidant; on the other hand, the coating can have a slow‐release effect on CEO and prolong its action time (Chang et al. 2023). In meat packaging, CEO nanoemulsion can fully contact the food surface, inhibit the growth of *E. coli* and *S. aureus* , which are common in meat, and maintain the color, flavor, and texture of meat (Santos Pimentel et al. 2024). For example, Chuesiang et al. (2023) explored the effect of cinnamon bark oil nanoemulsion (CBON) on the sensory properties of Asian seabass refrigerated fillets. CBON with a droplet size of 50.71 nm was prepared by the phase transition temperature method. Comparison with ordinary emulsion (BCBO) revealed that CBON could significantly mask the fishy odor, delay the degradation of cinnamaldehyde, and reduce the generation of oxidation products such as benzaldehyde. Sensationally, CBON avoided the yellowing and strong odor of fish fillets caused by BCBO, and the overall acceptability was higher than that of BCBO and the control group after 8 days of refrigeration.

The main preparation methods for NEs include HPM, ultrasonication, and low‐energy emulsification (e.g., PIT). Different emulsifiers, encapsulation methods, and oil phase compositions significantly affect the physicochemical properties of the CEO delivery system (Panwar et al. 2024). As shown in Table 1, by varying the emulsifier, encapsulation method, and the ratio of CEO to medium‐chain triglycerides (MCT) in the oil phase, the prepared delivery systems showed significant differences in key parameters such as average droplet size and zeta potential. These characteristics are directly related to the stability and functionality of the CEO delivery system in subsequent applications and provide basic data for further optimization of the system design.

**TABLE 1:** Physicochemical properties of CEO nanoemulsions with different emulsifiers and preparation conditions.

Emulsifier	Method	Oil phase	Average droplet size (nm)	Zeta potential (mv)	References
Tween 20, Span 80	Ultrasonic emulsification, PIT	25% (w/w) CEO	200.3 ± 4.71	−20.0 ± 0.81	Özakar et al. (2024)
HPCD/LAE inclusion complex	HPM	3% (v/v) CEO+4% (v/v) MCT	96.71 ± 0.43	—	Xing et al. (2024)
Tween ‐ 80, HPCD	Ultrasonic emulsification	1% (v/v) CEO	133.43	—	Hou et al. (2021)
Tween 80	PIT	40% (w/w) CEO+60% (w/w)	50.71 ± 1.48	—	Chuesiang et al. (2020)
Tea saponin	Ultrasonic emulsification	3% (v/v) CEO	169.6 ± 1.1	−36.2 ± 0.9	Zhu et al. (2025)
Tween 80	Ultrasonic emulsification	2% (v/v) CEO	66.14 ± 0.37	−6.27 ± 0.26	Jannah et al. (2025)

Abbreviations: HPCD, hydroxypropyl‐β‐cyclodextrin; LAE, lauryl arginine ethyl ester; MCT, medium‐chain triglycerides; PIT, phase transition temperature.

#### 2.1.3 Pickering Emulsions

Pickering emulsions (PEs), as colloidal systems stabilized by solid particles, form stable emulsions by adsorption of nanoscale solid particles at the oil–water interface, exhibiting excellent anti‐agglomeration and anti‐Ostwald ripening stability compared to conventional surfactant‐stabilized emulsions (Wang, Yang, et al. 2024). In CEO encapsulation, the construction of PEs relies on the interfacial adsorption of natural polymer nanoparticles, such as zein‐pectin composite nanoparticles, cellulose nanoparticles, chitosan‐gum arabic cohesive complex, cellulose nanocrystals, and so on. These solid particles bind to the CEO through electrostatic interactions, hydrogen bonding, or hydrophobic interactions to form a stable oil–water dispersion system, which forms a physical barrier to inhibit droplet aggregation.

The stability of PEs is closely related to the surface charge, hydrophilicity, and interfacial adsorption density of solid particles. For example, the adsorption of zein nanoparticles at the oil–water interface relies on the hydrophobic interaction of its hydrophobic amino acid residues with the CEO to form stable emulsion droplets (Wang, Li, et al. 2024). The interfacial interaction not only ensures the physical stability of PEs but also optimizes the antimicrobial efficacy by regulating the release rate of CEO. Yang et al. (2024) first prepared zein‐gallic acid covalent complexes as emulsifiers through alkaline treatment to stabilize CEO and form PEs, and then blended them with chitosan to prepare chitosan‐based films loaded with CEO PEs. Experiments showed that when 15% (v/v) of CEO Pickering emulsion was added, the mechanical and barrier properties of the films were significantly improved, while the light transmittance and thermal stability were enhanced. In addition, the loading of the emulsion improves the antioxidant activity of the film, resulting in high antimicrobial performance against food pathogens, and the slow‐release behavior of the CEO effectively prolongs the bioactivity of the film.

According to the difference of energy required in the preparation of PEs, the preparation methods can be classified into two categories: low‐energy and high‐energy methods, such as mechanical stirring, ultrasonication, HPM, microfluidization, membrane emulsification, etc. (Wu, Wang, et al. 2024). Low‐energy methods have the advantages of economy and simplicity of operation, and emulsification is achieved by regulating the oil–water interface and surfactants (Chen, Zhong, et al. 2025). High‐energy methods rely on equipment such as ultrasonic machines, high‐pressure homogenizers, etc., which utilize mechanical energy with limited efficiency and are costly. However, the high‐energy method is preferred by industry because it usually uses relatively few surfactants. Yu et al. (2024) used zein‐citrus pectin nanoparticles to stabilize CEO to prepare Pickering emulsion and constructed edible films (PEF) by the cast film‐forming method. It was found that PEF exhibited two‐sided differential wettability due to the spatial distribution of emulsion microdroplets, with the upper surface being partially hydrophobic and the lower surface being hydrophilic, and the mechanical properties (30%–50% increase in tensile strength) and transparency were superior to those of direct emulsion film. Its independent micro‐droplet structure enhances essential oil loading (up to 8.3%) and slow‐release effect, with an antibacterial rate of more than 90% and biodegradable properties, providing a new strategy for environmentally friendly food packaging, especially for greasy food leakage prevention and antibacterial preservation.

The application of PEs in food‐active packaging is mainly realized by combining them with biopolymer films/coatings. For example, Zhang, Guo, et al. (2024) successfully enhanced the stability and functionality of the corn alcohol‐soluble protein‐gum arabic‐cinnamon bark oil Pickering emulsion system by constructing it. It was shown that the 0.8% gum arabic‐modified emulsion would result in a more uniform particle size distribution and a more stable zeta potential while maintaining the antioxidant/antimicrobial properties.

### 2.2 Nanocarriers

Encapsulation or modification of CEO using nanotechnology can significantly improve the dispersion, stability, and target release efficiency of CEO. Nanomaterials, as auxiliary carriers, can enable CEO to better penetrate deep into the interior of the bioepidermis to exert antimicrobial effects and improve the mechanical properties, specific surface area, and biological activity of the biopolymeric membrane (Xu et al. 2024). Specifically, the nanoscale size will accelerate the penetration and disruption of CEO into microbial cell membranes and improve the antibacterial activity. Meanwhile, the protective effect of the polymer matrix inhibits the oxidation of the CEO. Currently, the mainstream nanocarriers include types of nanoliposomes, nanogels, nanoparticles, nanosponges, NEs, and nanofibers (Zhang, Lin, et al. 2024).

Nanosponges (e.g., cyclodextrin polymers) physically capture CEO molecules through porous structures, effectively masking pungent odors and reducing volatile loss (Kaur et al. 2021). Pant and Bhattacharya (2024) prepared α/β‐cyclodextrin nanosponges loaded with cinnamon extract/trans‐cinnamaldehyde by the freeze‐drying method using a molar ratio of α/β‐ cyclodextrin in the ratio of 1:6 and carbonyldiimidazole as the cross‐linking agent. β‐Cyclodextrin nanosponges showed significantly higher encapsulation efficiency of trans‐cinnamaldehyde and cinnamon extract than α‐cyclodextrin nanosponges. The cyclodextrin nanosponges loaded with cinnamon extract showed stronger antioxidant activity. The antimicrobial activity of the material loaded with trans‐cinnamaldehyde was superior, with a circle of inhibition of 47.66 ± 0.51 mm against *Pseudomonas aeruginosa* . Nanogels are three‐dimensional networked nanoparticles formed by physical or chemical cross‐linking of hydrophilic polymers. In CEO encapsulation, they utilize their unique swelling properties and environmental responsiveness to embed CEO molecules into the polymer network pores through hydrophobic interactions or hydrogen bonds, forming stable complexes. This process inhibits the oxidation and volatilization of CEO, while its hydrophilic matrix improves the dispersibility of CEO in aqueous phases. Rao et al. (2025) evaluated the potential application of phosphorylated egg white protein (P‐EWP) nanogels prepared by three methods—microwave‐induced phosphorylation modification, gelation treatment, and ultrasonic nanotechnology—as a CEO delivery system. Experiments showed that the P‐EWP‐CEO nanogel had a uniform particle size distribution and was highly stable under high temperatures (90°C) and ionic strength (200 mM NaCl) conditions. The nanogel exhibited long‐lasting bacteriostatic activity against *E. coli* , *S. aureus* , and *Listeria monocytogenes* with hydrophobic interactions and disulfide bonds as the main intermolecular forces.

#### 2.2.1 Nanoparticles

Nanoparticles, as an emerging functional material, are nanoscale (1–1000 nm) particulate systems formed by encapsulating or loading target substances using carriers such as natural or synthetic polymeric materials, lipids, and inorganic materials (e.g., silica) (Muhammad et al. 2019). The stability of nanoparticle systems mainly depends on the synergistic effect of surface charge and spatial site resistance. The structure of nanoparticles is diversified, including core‐shell, multilayer vesicles (e.g., liposomes), and porous networks (e.g., mesoporous silica), etc. The stability, controlled release, and bioactivity of the target substances can be significantly enhanced by structural design, and they can be applied to food preservation, antibacterial packaging, etc. (Qin et al. 2024). López‐Cano et al. (2023) investigated the potential of different nanoparticles (TiO_2_, CaCO_3_, and Al_2_O_3_), considering both their pure forms and those modified with CEO. Al_2_O_3_ nanoparticles exhibited the strongest interaction with CEO, resulting in an approximately 40% increase in antioxidant capacity and the conferral of antimicrobial properties, particularly against Gram‐negative bacteria. In contrast, TiO_2_ and CaCO_3_ nanoparticles exhibited limited interaction with CEO, resulting in lower antioxidant capacity and antibacterial activity. Experimental results indicated that incorporating CEO into metal oxide nanoparticles enhances their antioxidant and antibacterial effects while improving the mechanical and thermal properties of PLA films.

Nanoparticle loading of CEO can effectively delay the volatile loss of CEO and confer targeted release properties, which are effective in fresh‐cut fruits and vegetables, meat, bakery products, and other areas of preservation applications. Bakr et al. (2024) investigated the inhibitory effect of CEO (3.125 μL/mL) and CEO‐NPs (1.56 μL/mL) on Aspergillus flavus. It was shown that the number of Aspergillus flavus increased in the experimentally contaminated beef burger control and decreased in the samples spiked with CEO, while no Aspergillus flavus was detected in the samples spiked with CEO‐NPs at the 9th day of refrigerated storage. Nanoparticles also have adjustable particle size, surface properties, and release characteristics, allowing them to be precisely tailored to the specific needs of food packaging (Jiang et al. 2020). Notably, surface modification of nanoparticles can further optimize their dispersion in the aqueous phase; e.g., carboxymethylcellulose‐coated nanoparticles can enhance the aqueous solubility of CEO (Tan et al. 2024).

Polymeric nanoparticles, as a typical representative of nanoparticles, are based on natural polymers (chitosan, sodium alginate, and whey proteins) or synthetic polymers (PLA and PVA), which have the advantages of being highly tunable and stabilizable (Hong et al. 2024). Martínez‐Aguilar et al. (2023) developed an active packaging film. The film was prepared using PLA and SiO_2_ nanoparticles (NPs, 0.1% w/w) chemically modified by CEO. The modified SiO_2_ nanoparticles were shown to have high 2,2‐diphenyl‐1‐picrylhydrazyl (DPPH) radical inhibition, good thermal stability, and strong *E. coli* inhibition.

Polymer nanoparticles can reduce the adsorption of odor molecules on the surface by controlling the particle size, and the commonly used preparation techniques include nanoprecipitation, emulsification‐solvent evaporation, ionic gelation, HPM, and ultrasonication (Sonar et al. 2023). The preparation technique has a significant effect on the physicochemical properties, such as particle size, zeta potential, and encapsulation rate. Nanoprecipitation is the rapid injection of an organic phase into an aqueous phase to initiate polymer precipitation, and the particle size is affected by the difference in solvent polarity and injection rate (Bovone et al. 2022). The emulsification‐solvent evaporation method involves the dispersion of an oil phase in an aqueous phase containing an emulsifier, followed by the evaporation of the organic solvent to form nanoparticles, with the key factors being the type of emulsifier and the rate of agitation (Li et al. 2021). The ionic gelation method is to form a gel network through the interaction between anions and cations, in which the CEO is encapsulated, and the particle size is affected by the ion concentration and pH value. For example, chitosan and sodium alginate can undergo ionic reactions to form gel beads under certain conditions. The interaction of CEO with these charged polysaccharides immobilizes them in the gel network, thus achieving a slow‐release effect, as well as improving the thermal and antioxidant stability of essential oils and prolonging their duration of action in foods (Patiño‐Ruiz et al. 2020). The HPM method relies on high shear to break lipid particles to form nanoparticles, and the key controlling factor is the homogenization pressure. The ultrasonication method is to refine the particle size by the ultrasonic cavitation effect, and the key factor of particle size is the ultrasonication time.

#### 2.2.2 Nanofibers

With their high specific surface area (e.g., 50 m^2^/g), high porosity, high loading rate, and excellent mechanical properties and air permeability, nanofibers show unique advantages in the field of food‐active packaging (Mohammad et al. 2024). When used as a packaging liner or coating material, the nanoscale network structure can realize food preservation through the dual mechanism of physical barrier and active release. On the one hand, the dense pore network of nanofibers can effectively resist the intrusion of external environmental factors such as oxygen and moisture; on the other hand, CEO can inhibit microbial growth and block the moldy process of food (Xia et al. 2023). Li et al. (2025) encapsulated CEO‐loaded dealkalized lignin (DAL)‐stabilized emulsions in PVA and zein by using emulsion electrostatic spinning technology. The antimicrobial activity was higher compared with DAL/CEO emulsion and pure CEO. The nanofiber membrane was shown to exhibit a low water vapor transmission rate, which helps to reduce the transfer of moisture from the external environment to the interior of the package. In addition, the nanofiber membrane exhibits strong antioxidant activity, neutralizing 94.54% of DPPH free radicals. The nanofiber film also showed strong antimicrobial effects, with inhibition circles of 19.47 and 30.37 mm against *E. coli* and *S. aureus* , respectively.

Packaging materials made of nanofibers are flexible, and their most significant advantage is that they can be processed non‐thermally, thus enabling the encapsulation of many heat‐insensitive active substances (Arabian et al. 2025). A wide variety of polymers are used to prepare nanofibers, such as gelatin, cellulose, chitosan, PVA, PLA, polycaprolactone (PCL), etc., which endow the nanofibers with certain mechanical and barrier properties (Akpınar et al. 2025; K et al. 2024). Chen, Zhang, et al. (2024) used electrospinning to prepare antibacterial fiber membranes loaded with cinnamaldehyde using materials such as gelatin and corn alcohol‐soluble protein. The prepared nanofibers had smooth surfaces without beading, with an average diameter ranging from 1.46 to 2.02 μm. Compared to untreated strawberries, fiber membranes containing 15 wt% and 20 wt% cinnamaldehyde extended the shelf life of strawberries to 6 days.

Studies have shown that the performance of films used in active packaging is highly dependent on the manufacturing process and that the unique structure of nanofiber films provides faster release rates of active compounds compared to the dense structure of conventional films (Lin et al. 2022). The structural design of nanofibers can modulate the CEO release behavior. For example, core‐shell nanofibers (e.g., PLA skin wrapped around a CEO/β‐CD core) can achieve slow release of CEO by controlling the thickness of the shell (Liu et al. 2017).

**FIGURE 1:**
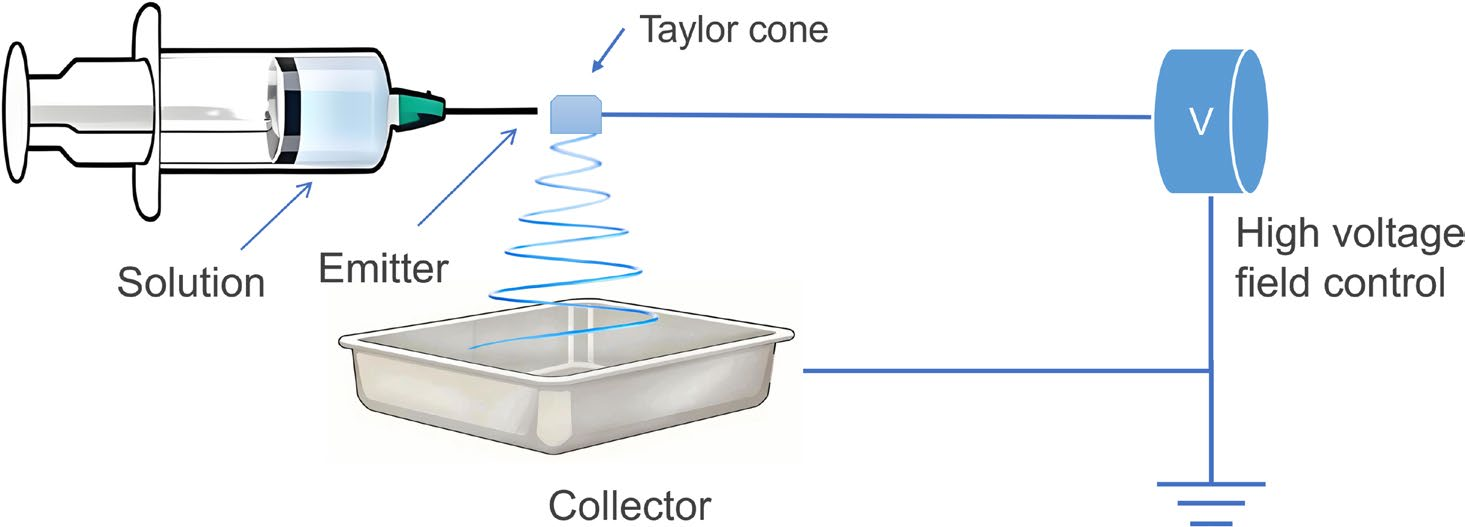
Schematic diagram of an electrospinning device.

Electrostatic spinning technology, as an efficient method of preparing nanofibers, is widely used in the fields of materials science and biomedicine (Maliszewska and Czapka 2022). Figure 1 shows a typical electrostatic spinning device: the solution injector stored in the polymer solution and controlling the flow rate delivery, the emitter (needle) connected to a high‐voltage power supply, the voltage at the tip of the emitter forming a strong electric field so that the solution is charged and the formation of a Taylor cone, and the formation of jets under the action of the strong electric field. The jet in the electric field is driven by the electric field force for stretching and refinement, while the solvent volatilizes, and ultimately by the collector to undertake curing, forming a high specific surface area and adjustable pore structure of the fiber membrane or fiber mat (Huang et al. 2024). The device induces the solution jet to undergo “whip instability” (illustrated by the helical trajectory in the figure) by electric field force, which causes the jet to rapidly split and fibrillate and ultimately deposit as nanofibers. By adjusting the solution formulation (polymer type, concentration), electric field parameters (voltage, distance), and environmental conditions (temperature, humidity), the diameter, morphology, and mechanical properties of nanofibers can be precisely controlled to meet the diverse application requirements of CEO. In addition, process parameters such as solution viscosity, flow rate, conductivity, nozzle‐collector distance, etc., also affect fiber quality (Li, Li, et al. 2024). For example, if the voltage is too high, beading will occur if the fiber diameter is not evenly distributed. If the receiving distance is too close, the fibers will not be able to dry and cure in time and will stick together, which will affect the fiber morphology and properties (Jiao et al. 2024).

During the electrospinning process, there are interactions between CEO and polymer molecules, such as physical entanglement and chemical bonding, which enable the essential oils to be uniformly dispersed inside the fibers or adsorbed on the surface (Shahbazi et al. 2024). The nanofiber membrane formed has a porous structure that enables the slow release of essential oils and prolongs their antimicrobial and antioxidant effects, as well as improves the breathability and moisture resistance of food packaging (Wang and Su 2024). Xiang et al. (2024) developed an antimicrobial packaging material doped with CEO by electrostatic spinning using PVA and corn alcohol‐soluble protein. Experiments showed that the encapsulation rate and loading of CEO reached 87.42% and 13.83%, respectively. The average diameter of the nanofilm was 976.12 nm, and the circles of inhibition against *E. coli* , *S. aureus* , *Pseudomonas fluorescens* , and *L. monocytogenes* were 8.41, 26.77, 18.93, and 36.24 mm, with a non‐contact inhibition rate of 100%, respectively. The nanomembranes effectively extended the shelf life of pork tenderloin and shrimp to 10 and 6 days, respectively, with 90.9% and 80.4% release of CEO, respectively.

### 2.3 Molecular Inclusion

Molecular encapsulation technology uses biomolecules with cavity structures as carriers to achieve the encapsulation of bioactive ingredients, which is suitable for improving the dispersion of CEO in aqueous matrices (Silva et al. 2024). Different types of cyclodextrins (e.g., α‐, β‐, and γ‐cyclodextrins) have structural differences that affect the binding and molecular interactions during the encapsulation process. For example, the unique channel structure of γ‐cyclodextrin (γ‐CD) enables it to encapsulate hydrophilic molecules, which is advantageous in protecting the active ingredient from the environment, reducing odor release, and realizing controlled release, etc. Phan, Tran, et al. (2024) investigated the effect of CEO and γ‐cyclodextrin in papaya fruit preservation by preparing an inclusion complex with γ‐cyclodextrin. At a dose of 10 g/kg, the inclusion‐treated fruits showed a decrease in disease severity index and a 55% increase in shelf life compared to untreated fruits.

The enhancement of essential oil properties by molecular inclusion complexes is manifested in several ways: it improves the water solubility of CEO, masks its strong odor, and reduces volatilization losses, as well as enhances the stability of CEO during food processing and storage (Dodero et al. 2021). For example, Sun et al. (2018) explored the encapsulation mechanism of cinnamaldehyde with β‐CD, hydroxypropyl‐β‐cyclodextrin (HP‐β‐CD), and dimethyl‐β‐cyclodextrin (DM‐β‐CD) through a combination of molecular simulations and experiments. It was shown that the benzene ring of cinnamaldehyde was inserted into the hydrophobic cavity of cyclodextrin to form a 1:1 inclusion complex, among which DM‐β‐CD was the most hydrophobic due to the methyl modification, with a binding energy of 60.48 kJ/mol, which increased the solubility of cinnamaldehyde from 0.675 to 21.127 mM, with a dissolution rate of more than 90% in 40 min. Experimental verification (1H NMR, 2D—ROESY, infrared spectroscopy) was consistent with molecular dynamics simulations, revealing that hydrophobic interactions and hydrogen bonds were the main driving forces.

The β‐cyclodextrin (β‐CD) consists of seven glucose units linked by α‐1,4 glycosidic bonds to form a cyclic oligosaccharide, which is surrounded by hydrophilic groups on the outside and forms a hydrophobic cavity on the inside, and this structure allows it to encapsulate the CEO through subject‐object interactions (Wen et al. 2016). The cinnamaldehyde molecule first diffuses into the hydrophobic cavity of β‐CD driven by hydrophobic interactions, and then its aldehyde group forms hydrogen bonds with the hydroxyl group at the β‐CD port and finally forms a stable encapsulated structure through non‐covalent interactions such as van der Waals forces (Zou et al. 2021). This inclusion process involves various intermolecular forces, such as hydrogen bonds, dispersion forces, van der Waals forces, electrostatic forces, and hydrophobic forces.

Different preparation methods can affect the inclusion effect (Herrera et al. 2019). For example, the saturated aqueous solution method promotes the precipitation of inclusion complexes from a saturated solution by adjusting external conditions. This method is simple to operate but time‐consuming and temperature‐sensitive and is primarily suitable for small‐scale production (Wang et al. 2019). The grinding method promotes inclusion through dry grinding, which does not require solvents but has a low inclusion rate, making it suitable for inclusion of heat‐sensitive substances (Cabrera Quiñones et al. 2023). The ultrasonic method utilizes cavitation effects to achieve encapsulation, reducing reaction time but with higher equipment costs (Li, Lv, et al. 2022). The spray‐drying method mixes essential oils with wall materials, atomizes them, and dries them into powders. The type and proportion of wall materials influence microcapsule morphology and particle size, thereby affecting encapsulation efficiency and release characteristics (Rivera et al. 2024).

In the application of food packaging and preservation, the inclusion has the advantages of masking odor, enhancing solubility, and improving stability. For example, Guan et al. (2024) investigated the construction of microencapsulated inclusion with β‐CD as wall material and CEO as core material and then loaded the inclusion into Polyvinylidene Fluoride (PVDF) nanofiber membranes by electrostatic spinning technology. The results showed that the membrane had high inhibitory activity against *E. coli* and *S. aureus* and could be used to extend the shelf life of pork, which has potential application in the field of food preservation in high humidity. Phan, Do, and Thuong (2024) prepared an inclusion compound using β‐CD and CEO in which the amount of β‐CD was 1.0 mmol (1.13 g), and the amount of cinnamic aldehyde added was about 1.5 mmol in a volume of 3.0. It was confirmed that when the dosage of CEO‐β‐CD inclusion compound was 2.5 mg per gram of mango, the overall quality of mango could be significantly improved. However, the inclusion technology still faces challenges in large‐scale application. For example, the low solubility of cyclodextrins in water may limit their potential to deliver active ingredients in aqueous media. Organic solvents used in the inclusion process (e.g., ethanol or ether) may reduce loading due to insufficient solubility of the active ingredient. In addition, factors such as size matching of the cyclodextrin cavity to the active ingredient and functional group interactions also affect the encapsulation efficiency. Future studies could enhance the water solubility of cyclodextrins by modifying them (e.g., using HP‐β‐CD, nanoscale β‐cyclodextrins) or enhance the utility of β‐CD for delivering CEOs by complexing it with other biopolymers (Shu et al. 2025).

### 2.4 Microcapsules

Although molecular inclusion can improve the water solubility and stability of CEO, its microencapsulation application is still limited by the insufficient water solubility of cyclodextrins and easy precipitation (Riós Pérez et al. 2024). Microencapsulation technology uses natural or synthetic polymers as the wall material (such as gelatin, starch derivatives, chitosan, gum arabic, etc.) and wraps the CEO by constructing a core‐shell structure to realize the isolation and protection of the active ingredient (Noghabi and Molaveisi 2020). The mechanisms of microencapsulation to enhance the efficacy of CEO include a physical barrier to inhibit the oxidation of CEO, controlled release to prolong the antimicrobial efficacy, and wall protection to maintain the chemical stability of CEO (Hu et al. 2025). This technology can reduce the loss of CEO by volatilization, extend the duration of action, and reduce its negative impact on the physical properties of packaging materials. Specifically, in the early stage of storage, microcapsules can block environmental factors to prevent the rapid dissipation of essential oils, and as humidity rises or temperature fluctuates, the wall structure gradually breaks down, and CEO continues to play its antimicrobial and antioxidant roles through the mechanism of slow release (Liu, Wang, et al. 2023). In addition, the particle size effect of microencapsulation (micron level) avoids the controversy over the biosafety of nanomaterials and makes it easier to pass the certification of food regulations, which provides technical support for the large‐scale application of CEO in food preservation and packaging. However, the microencapsulation process is relatively complex and costly, and the quality indexes, such as particle size and encapsulation rate of microcapsules, need to be strictly controlled.

Methods for the preparation of microcapsules include spray drying, freeze drying, complex coacervation, ionic gelation, precipitation, and inclusion complexation (Chen et al. 2023; Osanloo et al. 2023). The release characteristics of microcapsules are closely related to the wall structure. Kean et al. (2022) used four methods for microencapsulation of CEO to determine the suitable method for the preparation of CEO microencapsulated powder. Experiments showed that the powder recovery rate of spray‐drying was much lower than the other methods. Freeze‐drying gave the highest encapsulation rate (92.3%–95.2%), followed by drum drying, spray drying, and manual methods. CEO microencapsulation significantly inhibited bacterial growth in a sample of minced chicken meat refrigerated for 12 days.

In industrialized applications, the spray‐drying method dominates with simple operation and high production efficiency. In this method, the CEO is mixed with the wall material solution to make a homogeneous emulsion, which is sprayed into a stream of hot air through a spraying device so that the solvent evaporates rapidly and the wall material is cured to form microcapsules (Noghabi et al. 2023). However, high temperatures may destroy the thermosensitive components, and the particle size distribution and encapsulation rate may be affected by a variety of preparation factors (Lai et al. 2023). For example, Zhao et al. (2024) prepared CEO microcapsules using chitosan hydrochloride (CHC) and gelatin (GE) as wall materials under the conditions of an inlet air temperature of 180°C and a GE:CHC ratio of 10:1. The experiments showed that the microcapsules successfully encapsulated CEO, retained the volatile components, and had good thermal stability, which could significantly extend the shelf life of bread and inhibit the growth of dominant spoilage bacteria such as Aspergillus spp.

The freeze‐drying method first freezes the emulsion and then sublimates and dehydrates it under vacuum. The freeze‐drying method, although better able to maintain the activity of the CEO, is more costly and requires a longer processing time. Baghi et al. (2023) selected two plant‐based emulsifiers (soy lecithin and pea isolate protein) to compare the emulsification and encapsulation ability of trans‐cinnamaldehyde treated by both spray‐drying and freeze‐drying techniques. The average particle sizes of the spray‐dried (SD) and freeze‐dried (FD) powders were 8.35 and 144.49 μm, respectively, and the encapsulation efficiencies (EEs) of soy lecithin and pea isolate protein in the SD powders were similar, with an average value of 95.69%. The EE of FD powders was generally lower than that of SD powders, with an average EE of 58.01% for lecithin‐containing powders and 83.93% for pea protein‐containing powders. However, the water content of FD powder (2.83%) was lower than that of SD powder (4.72%). Both NEs and dried powders showed significant bacteriostatic activity against *E. coli* and *Listeria innocua* .

Complex coacervation is a process where wall materials dissolved or dispersed in a solution undergo physical and chemical reactions to induce coagulation, thereby encapsulating CEO inside to form microcapsules. The method utilizes electrostatic interactions between oppositely charged polysaccharides and proteins to form a cohesive system. CEO in this system, the interactions between the charged particles promote the essential oils to be encapsulated inside the cohesive material (Muhoza et al. 2022). Factors such as pH and ionic strength during the coalescence process affect the coalescence effect and the encapsulation rate of essential oils. Liu, Zhao, et al. (2023) prepared CEO antimicrobial microcapsules using the complex coalescence method, choosing chitosan quaternary ammonium salt (HACC) and gum arabic (GA) as the encapsulation materials. The optimal conditions for the preparation of CEO microcapsules were determined by response surface methodology, with a core‐to‐wall mass ratio of 1:1, a pH value of 4.5, and a CaCl_2_ mass concentration of 0.7 wt%, at which time the microcapsule actual encapsulation rate reached 90.72% ± 1.89%. The CEO microcapsules exhibit a spherical shape with an average particle size of 6.31 μm. The minimum and maximum release rates within 30 days are 19.66% and 49.79%, respectively.

The precipitation method is to precipitate the wall material in the solution and wrap the CEO by means of adjusting the pH of the solution, temperature, adding precipitant, etc. Complex coacervation and precipitation methods are relatively low cost and can be operated at room temperature but need to accurately control pH and ionic concentration and may require the use of toxic and hazardous chemicals in the preparation process, which will have a certain impact on the environment. The inclusion complexation method utilizes the interaction between the compound and the CEO to form a stable complex, and then the microcapsules are formed through the coating of the wall material. This method can significantly improve the stability of CEO, but the preparation process is more complicated, and the encapsulation rate may be limited by the stability of the complex (Liu et al. 2024). Li et al. (2020) used three processes, namely, saturated aqueous solution, molecular embedding, and ultrasonication, respectively, to prepare CEO microcapsules using Highland barley starch (HBS) as the wall material. HBS has good encapsulation ability and excellent biocompatibility and thermal stability due to its multi‐hydroxyl structure forming hydrogen bonds with CEO. Experiments showed that the microcapsules prepared by the molecular embedding method had the best performance, with an encapsulation efficiency of 88.2%, a yield of 79.1%, a release rate of 11.5% after 25 days of storage, and a smooth and dense microstructure, which effectively delayed the release of CEO.

Microcapsules prepared by the Pickering emulsion template method have the advantages of good monodispersity, uniform particle size, and environmental friendliness. However, they have stringent requirements for particle selection and preparation, wall formation requires precise control of conditions, and it is difficult to completely remove residual particles during post‐processing (Liu, Wei, et al. 2023). Li, Gao, et al. (2022) prepared composite microcapsules by microencapsulating CEO through the Pickering emulsion template method using Pickering emulsion stabilized based on SiO_2_ nanoparticles. The microencapsulation technique effectively enhanced the stability of CEO and achieved slow release of loaded essential oils. The microcapsules showed a minimum inhibitory concentration of 2 mg/mL against *E. coli* and *S. aureus* , which provided a long‐lasting antimicrobial effect.

**FIGURE 2:**
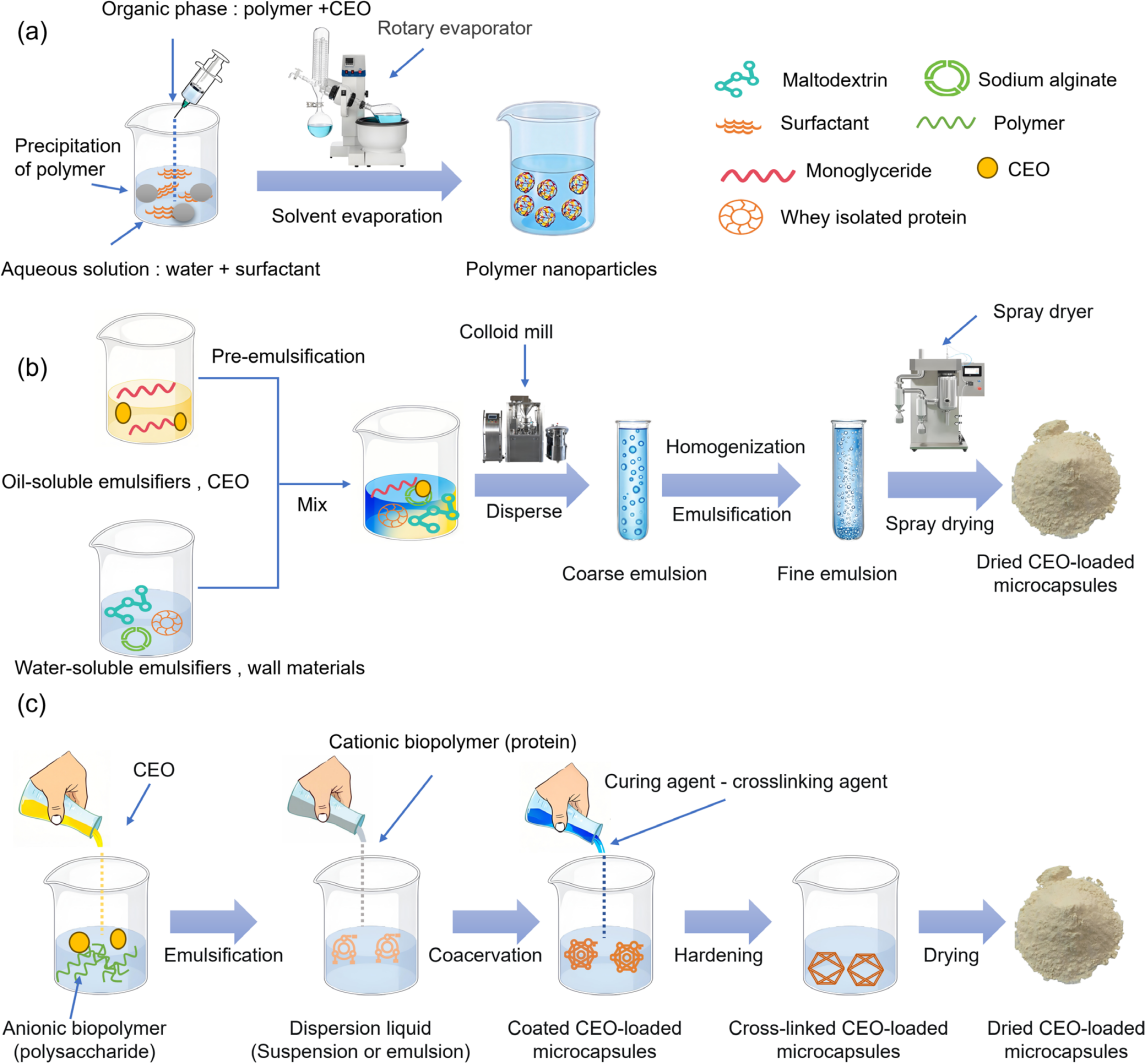
Three methods for CEO microencapsulation: (a) preparation technique of polymer nanoparticles based on solvent evaporation method; (b) emulsification‐spray‐drying method; (c) biopolyelectrolyte microencapsulation technique based on complex coacervation and chemical cross‐emulsification‐spray‐drying method.

Figure 2 illustrates three key technological pathways widely used in the preparation of CEO microencapsulation. The core objective is to encapsulate volatile and unstable CEO inside a protective wall material to significantly enhance its stability, mask undesirable flavors, control the release rate, and expand its forms of application. Figure 2a depicts a preparation process based on O/W emulsions with solvent evaporation‐induced polymer precipitation, where an organic phase dissolved with hydrophobic polymers and essential oils is dispersed in a surfactant‐containing aqueous phase to form an emulsion, and the polymer precipitation is subsequently induced by the evaporation of organic solvent to encapsulate the essential oils to form nano‐ or micron‐scale solid particles. Figure 2b depicts the emulsion‐spray‐drying method, which employs a pre‐emulsified monoglyceride CEO mixed with an aqueous phase containing a composite wall material (maltodextrin/sodium alginate/whey isolate protein) and sucrose ester, homogenized to form a fine emulsion with droplet sizes and a uniform distribution of the O/W fine emulsions, and ultimately SD to instantly dehydrate and solidify the fine emulsions by spray drying, allowing the wall material to form a dense cladding layer around the oil droplets. Figure 2c depicts the microcapsule construction process based on electrostatic recombination of bio‐polyelectrolytes combined with chemical cross‐linking for hardening, which utilizes the self‐assembly of oppositely charged biopolymers (e.g., proteins and polysaccharides) on the surface of the oil droplet to form a primary wall layer, which is then cured by cross‐linking agents and ultimately, often, leads to the formation of a multilayered structured microcapsule.

### 2.5 Liposomes

Liposomes, as microscopic vesicular structures composed of phospholipid bilayers (particle size 50–1500 nm), can significantly improve the chemical stability and controlled release properties of CEO by encapsulating it in lipid bilayers or aqueous phase cores (Wang et al. 2021). Its biocompatibility, degradability, and low toxicity characteristics make it a promising delivery vehicle for food preservation. Phospholipid membrane fluidity can be regulated by cholesterol, but its phospholipid membrane is easily oxidized and needs antioxidant protection, which is suitable for targeting microbial membranes with high antimicrobial efficiency. Studies have shown that the antimicrobial activity and stability of CEO are significantly enhanced after liposomal encapsulation. For example, Farahmand et al. (2025) applied a hybrid protection system for encapsulating CEO to achieve higher protection of CEO in acidic beverage systems and to mask its strong odor and taste. Experiments were conducted to prepare loaded CEO‐liposomes with a particle size of 252.33 nm, ζ‐potential of 33.03 mV, and encapsulation efficiency of 81.40% in alginate microcapsules by optimizing the liposome formulation with a lecithin/cholesterol ratio of 5:1 and a wall/CEO ratio of 7.5 and combining them with microfluidic technology. Experiments showed that the encapsulation of CEO‐loaded liposomes using the liposome‐fluidic method significantly affected the release of CEO from the liposomes and successfully controlled the strong odor of CEO.

In food packaging applications, liposomes are often complexed with biopolymers to build functionalized membrane materials. For example, when liposomes are applied as food contact materials, their thermal stability remains limited. Therefore, a chitosan coating is applied to the surface of liposomes to enhance their thermal resilience. The gelatin composite membrane containing prepared chitosan‐CEO‐liposomes was shown to have an antimicrobial efficiency of 91.7% against *E. coli* O157:H7 and *Staphylococcus epidermidis* . In addition, the membrane helps to maintain the freshness and extend the shelf life of the food due to its antioxidant effect (Baek and Oh 2024).

Liposome encapsulation technology can effectively enhance the antimicrobial timeliness and functional durability of CEO in food systems by modulating their release kinetics and interfacial interactions. In addition, the synergistic design of liposomes with other nanocarriers further expands the application scenarios. The nanoliposomes utilize phospholipid bilayers to encapsulate lipophilic CEO, enhancing their water dispersion and delaying volatilization, while the similarity between phospholipid membranes and microbial cell membranes promotes targeted release. Wang et al. (2022) prepared cinnamaldehyde nanoliposomes (CIN‐NLs) by thin‐film dispersion and then modified them with ε‐ polylysine (ε‐PL), obtaining ε‐ polylysine/cinnamaldehyde nanoliposomes (ε‐PL/CIN‐NLs). It was shown that both CIN‐NLs and ε‐PL/CIN‐NLs had spherical nanoscale structures. After modification by ε‐PL (2 mg/mL), the particle size distribution of the nanoliposomes was enlarged and the encapsulation efficiency was improved. The highest encapsulation efficiency of ε‐PL/CIN‐NLs of 30.4% ± 0.2% was achieved when the CIN concentration was 4 mg/mL. Both CIN‐NLs and ε‐PL/CIN‐NLs showed significant inhibitory activity against the growth of Aspergillus flavus and Penicillium xanthophyllum. Emami et al. (2022) prepared CEO nanoliposomes by using a thin‐layer hydration–ultrasound technique. Three different co‐surfactants, glycerol, glyceryl triacetate, and propylene glycol, were used as the main component, and Tween 80 was used as a surfactant. The experiments showed that the nanoliposomes prepared with propylene glycol had the smallest average particle size (92.03 nm) and the largest net zeta potential (−24.1 mV) and were therefore selected as a more suitable co‐surfactant. The CEO nanoliposomes were prepared using propylene glycol as the co‐surfactant with different dosages of CEO (0.25, 0.5, and 1.25 mL). Although the antimicrobial activity after encapsulation was slightly lower than that of the free state, it still maintained efficient inhibition of *E. coli* and *L. monocytogenes* .

### 2.6 Performance Differences

CEO delivery systems differ widely in preparation principles, structural properties, and application efficacy. The loading rate of the inclusion is low, the controlled release mechanism is dependent on the host‐guest interaction, the stability is medium, and the cost is low. The advantage is the simplicity of preparation, and the disadvantage is that it is prone to burst release (Ahmed et al. 2022). NEs have a medium loading rate; the controlled release mechanism depends on surfactant modulation, medium stability, and medium cost. The advantage is high dispersibility, but it requires the use of large amounts of surfactants (Feng et al. 2023). Nanofibers have a high loading rate, the controlled release occurs through the physical barrier of the fiber network, and they have high stability but high cost. They have a high specific surface area but require specialized equipment for their preparation (Ding et al. 2023). Microcapsules have a high loading rate, and the controlled release mechanism relies on the degradation of the wall material. Advantages include compatibility with a wide range of wall materials, but the process is complex (Zhang, Yang, et al. 2023). PEs have a high loading rate, achieve controlled release through solid particle barriers, and exhibit extremely high stability. Their antimicrobial activity is strong, but the preparation of solid particles is a complex process (Ly et al. 2024).

The performance differences of various delivery systems are essentially determined by interfacial physicochemical properties. Nano/microemulsions rely on the molecular design of surfactants. PEs and nanoparticles focus on the interfacial engineering of colloidal particles, while microcapsules and molecular inclusion complexes emphasize the structural regulation of wall materials/carriers. For example, Wu, Zhang, et al. (2024) compared the effects of liposome and emulsion systems loaded with CEO on the quality and proteolysis of refrigerated minced pork. The CEO‐liposomes were prepared by the ethanol injection method, using phospholipids and cholesterol as wall materials to encapsulate CEO. The CEO‐emulsions, meanwhile, emulsified CEO through a chitosan‐pectin composite matrix with Tween 80 added as a surfactant. Experiments showed that the CEO‐liposomes were more effective in inhibiting microbial growth and delaying protein degradation, and their antimicrobial activity was enhanced by increasing the permeability of bacterial cell membranes, and they were more effective in protecting meat quality due to their small particle size (101.4 nm) and slow‐release stability.

## 3 Forms of Packaging

### 3.1 Active Packaging

The core value of active packaging, as a technology system that extends the shelf life of food by adding functional substances to the material and interacting with the environment inside the package, lies in the coupling of the activity of functional ingredients with the performance of the packaging material (Mu et al. 2023). CEO is rich in cinnamaldehyde and its derivatives, which can not only optimize the physical properties of the substrate by enhancing the mechanical strength and barrier performance of the packaging material but also enhance the efficiency of food preservation by virtue of the dual activity of antibacterial and antioxidant.

At the preparation level, the combination of CEOs with packaging substrates relies on direct addition, physical adsorption, and chemical bonding strategies (Ribeiro et al. 2024). For example, paper packaging is often impregnated or sprayed to attach CEO to the fiber surface. This method is relatively low‐cost, but it has problems such as fast evaporation of essential oils and poor compatibility with packaging materials. In addition, this method is also easy to affect the physical properties of packaging materials, such as making the paper less flexible and plastic transparent (López‐Gómez et al. 2020). On the other hand, polyethylene (PE), PVA, and other plastic packaging materials use high‐temperature and high‐pressure conditions during processing to realize the uniform dispersion of CEO inside the material.

**FIGURE 3:**
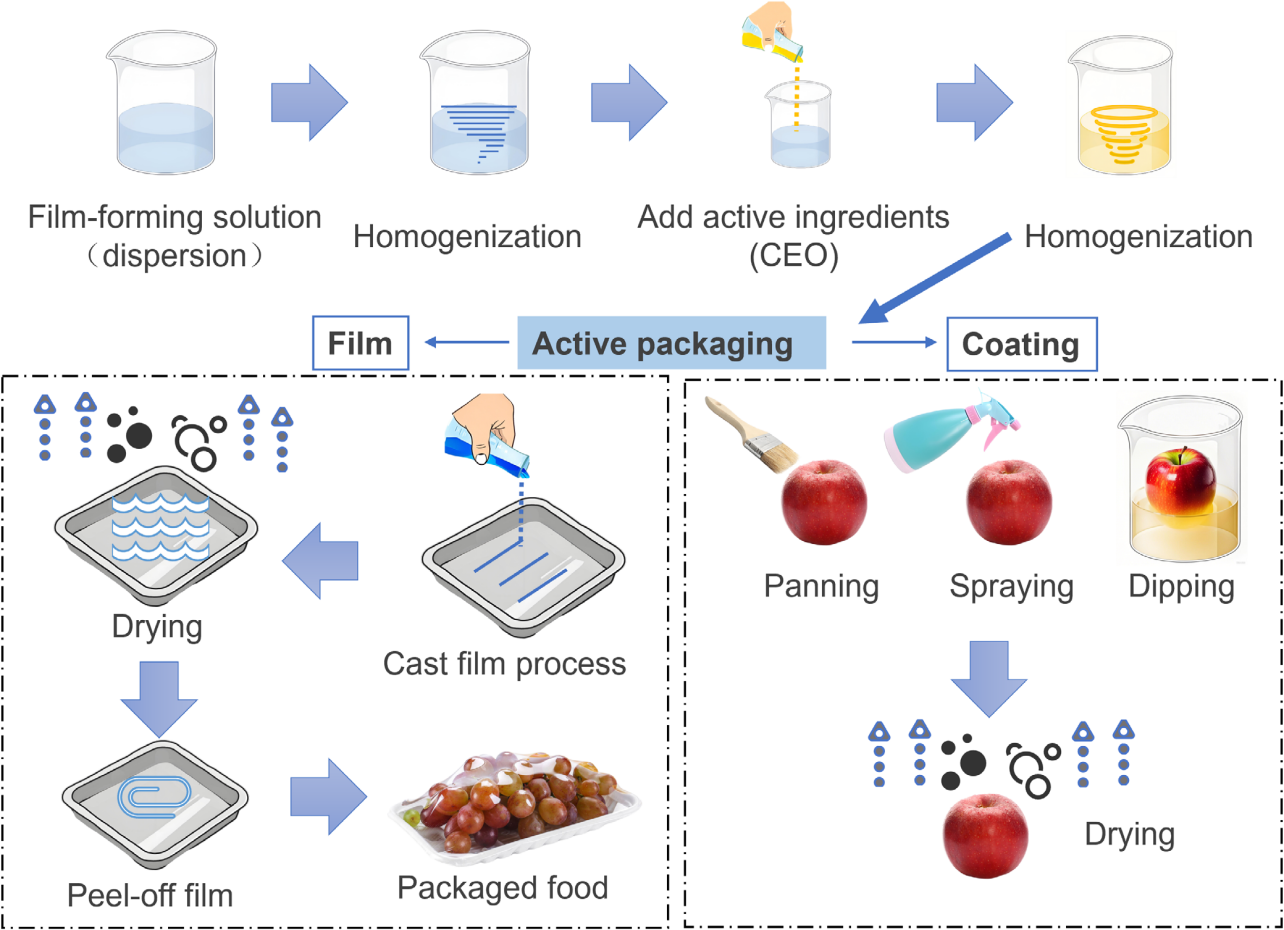
Flowchart of active packaging preparation process.

The common preparation process for active packaging begins with the formulation and homogenization of the film‐forming dispersion, which is then homogenized again after the introduction of the active component to ensure uniform dispersion (Figure 3). There are two technological paths in the molding stage: first, the dispersion is formed into a film by casting, which is dried, peeled, and applied directly to the food product. The second is to apply the dispersion directly to the surface of the food or packaging substrate by spin‐coating, spraying, or dip‐coating, and then drying and curing to form the active coating.

In food preservation applications, CEO‐containing active packaging maintains food sensory quality by regulating water migration and inhibiting enzyme activity and microbial growth (Devecioglu et al. 2022). For example, Hu et al. (2024) used hydroxypropyl‐β‐cyclodextrin/lauroyl arginine ethyl ester (HPCD/LAE) complex to emulsify CEO to prepare NEs, which were then complexed with maltodextrin (MD) to prepare HPCD/LAE/CEO/MD microcapsules by the spray‐drying method. The microcapsules were introduced into a starch/polyadipic acid butylene terephthalate base to prepare extruded blow‐molded films and used as strawberry freshness packaging materials. When the formulation was 4% HPCD/LAE, 3% CEO, and 10 MD, the microcapsules had the smallest particle size (3.3 μm), the highest CEO encapsulation rate (84.51%), and the best antimicrobial effect. The film can effectively reduce the weight loss rate, decay rate, and total colony and mold count of strawberries, thus prolonging the shelf life of strawberries.

**FIGURE 4:**
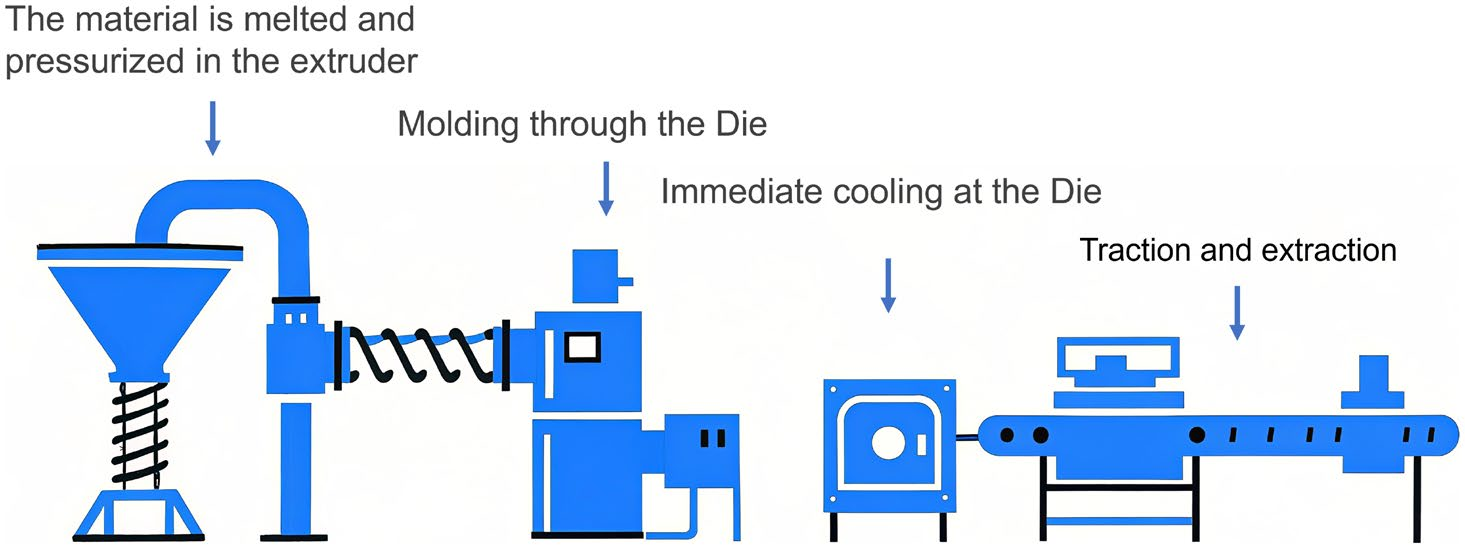
Schematic diagram of key stages in plastic extrusion process.

The molding of active packaging takes film as its main form, and its preparation process is divided into the casting method commonly used in laboratories and the extrusion method for industrial‐scale production. The casting method is the most commonly used film‐forming method in laboratory and pilot scale; the process is to dissolve the biopolymer in a suitable solvent and pour it into the mold, then remove the solvent to form a polymer film by hot air oven, tray dryer, etc. The film‐forming ability depends on the solubility of the polymer, and the drying process is crucial for the intermolecular interactions and the microstructure of the film (Suhag et al. 2020). The extrusion method is a widely used polymer processing method in industry, which utilizes the properties of thermoplastic polymers for molding through three steps: feeding, kneading, and heating. Plasticizers (e.g., polyethylene glycol) can be added during the molding process to improve film flexibility, although temperatures above the polymer's glass transition temperature need to be controlled (Lauer et al. 2018). The core process of continuous extrusion of plastics can be summarized as the melting and plasticizing of the material, the shaping and cooling of the die and curing, and the haul‐off of the final product (Figure 4). The process begins with an extruder where the solid plastic material is heated, sheared, melted, and pushed under high pressure. The melt then passes through a specially designed die to form the desired cross‐sectional shape and immediately enters the die cooling stage for initial cooling and shaping. Finally, the pre‐set product is pulled out by a pulling removal device at a rate precisely matched to the extrusion speed and removed from the line for subsequent cutting, winding, or stacking operations.

### 3.2 Edible Packaging

Edible packaging is a thin layer of edible biopolymers that forms a barrier on the surface of food to retard chemical deterioration and microbial contamination. Edible packaging is usually a packaging material made of natural biomolecules with functional ingredients (Sharma et al. 2022). They are mainly made of proteins, polysaccharides, and lipids. The chain interactions and hydrophilic/hydrophobic balance between the materials determine the barrier properties of the packaging against oxygen, moisture, and temperature (Trajkovska Petkoska et al. 2021).

Edible packaging is mainly in the form of edible coatings and films, which can be directly applied to the surface of food to form a barrier to water, gases, and flavor substances, maintaining the structural integrity of food (Jose et al. 2024). The preparation process of edible packaging (e.g., dip‐coating, spraying, etc.) significantly affects its film‐forming properties and adhesion effects. Among them, dip‐coating is widely used because of its ease of operation and uniform film formation, but the viscosity, density, and surface tension of the coating solution need to be precisely regulated.

As shown in Table 2, the edible packaging for encapsulating the CEO has significant effects in inhibiting microbial growth, reducing lipid oxidation, and maintaining sensory quality (Casalini et al. 2023). The application of encapsulated CEO to edible packaging significantly improves its uniform distribution properties in the packaging matrix compared to the direct addition of free CEO and prolongs the antimicrobial efficacy by controlling the release behavior. In addition, in the field of meat preservation, edible packaging containing encapsulated CEO can maintain the microbiological safety and nutritional quality of chicken, beef, and other products by inhibiting the growth of microorganisms such as *L. monocytogenes* and *P. aeruginosa* , as well as reducing the generation of off‐flavors (Morshdy et al. 2021). However, current edible packaging still faces challenges in large‐scale production, such as material compatibility, high‐temperature processing stability, and cost control. Parameters such as the loading of encapsulated CEOs, encapsulation efficiency, particle size, charge properties, and concentration all affect the antimicrobial activity and physicochemical stability of edible packaging (Ganeson et al. 2022). Future research needs to further optimize the synergistic effect of the encapsulation system and packaging materials and deeply investigate the interaction mechanism between encapsulated CEO and the food matrix in order to promote the wide application of edible packaging in the food industry.

**TABLE 2:** Effectiveness of CEO edible packaging/coating in food preservation.

Formulation	Dosage	Food	Main results	References
Coating	0.16 g	Orange	Enhanced barrier properties to inhibit the growth of Penicillium spp.	Erceg et al. (2025)
Coating	0.075 g	Pomegranate	Delayed release half‐life of CEO Inhibited *Escherichia coli* and *Staphylococcus aureus*	Arcot et al. (2024)
Coating	1200–2400 μL/L	Agaricus bisporus	Reduced weight loss and improved sensory quality Reduced respiration rate Maintained ascorbic acid and soluble protein contents	Golmohammadi et al. (2024)
Film	3% (v/v)	Love bite	Inhibited oxidation, metabolism, and microbial growth Maintained fruit firmness and reduced weight loss	Chen, Hu, and Zhang (2024)
Film	25 μL	Soft sliced bread	Inhibited Penicillium digitatum, yeast, molds, and fungal growth	Noshirvani et al. (2024)
Film	0.1% (v/v)	Red snapper fillets	Enhanced hydrophobicity and antioxidant activity Delayed spoilage and lipid oxidation of fish fillets (4°C, 8 days)	Zhao et al. (2022)

### 3.3 Intelligent Packaging

Intelligent packaging is a new type of system that can sense changes in the environment inside the package and provide feedback on the quality of food through color, fluorescence, and other signals. Its core value lies in transforming physicochemical parameters during food spoilage into visual signals and realizing the controlled release of CEO through dynamic changes in material structure (Li, Hu, et al. 2024). When food spoils, microbial metabolism (e.g., acid production by lactic acid bacteria, protein breakdown by *P. aeruginosa* ) leads to a decrease in the environmental pH, while tissue cell respiration and water migration cause an increase in humidity inside the package (Lin et al. 2023).

In humidity‐responsive systems, the release of CEO is triggered by moisture migration within the package. For example, Luo et al. (2022) developed poly(ethylene glycol)‐poly(caprolactone) (PEG‐PCL) micelles to encapsulate CEO, which triggered the rapid release of CEO by high humidity to make it dynamically responsive in mold‐prone environments. The hydrophilic segment of PEG‐PCL micelles, PEG, absorbs and swells in high‐humidity environments, which destroys the micelles' core‐shell structure and prompts the release of CEO encapsulated by a hydrophobic inner core, PCL. Experiments showed that the cumulative release rate of CEO reached 72% within 7 days at relative humidity (RH) ≥ 75%, which was significantly higher than that in low humidity environments (15% RH and 35% RH). In a strawberry preservation scenario at 85%–95% RH, the system resulted in a 59% reduction in spoilage and an increase in hardness retention to 23%. Lan et al. (2024) prepared humidity‐responsive antibacterial aerogels using chitosan and dialdehyde nanocellulose and loaded them with cyclodextrin‐cinnamaldehyde inclusion complexes. Experiments showed that the aerogel had a significantly higher cinnamaldehyde release rate at 98% RH than at 70% RH, which not only inactivated microorganisms such as *E. coli* and *S. aureus* but also absorbed exuded water from the produce through the absorbent mat function to prolong the strawberry shelf life.

As shown in Table 3, natural pigments are often used as indicators to achieve food freshness status monitoring in the field of food smart packaging, such as meat, fish, and strawberry foods. Anthocyanins are natural polyphenolic compounds that are extremely sensitive to changes in environmental pH. Under different pH conditions, anthocyanins can show different structures, including flavylium cation, pseudo‐base carbinol, quinone base, and chalcone, etc., and these different structures correspond to different colors (Zhou et al. 2024). When the food is fresh, its internal pH is in a specific range, and the anthocyanins in the film take on a color. As the food is stored, the pH of the food changes due to microbial growth and reproduction as well as the food's own metabolic activity. Specifically, at the fresh food stage, in an acidic environment (pH 1–4), anthocyanins are stabilized in the form of oxonium salts, and the film exhibits a pink color. At the stage of food spoilage, in a neutral to alkaline environment (pH 5–10), with the increase of ‐OH, the molecules deprotonate to form quinone bases, and the color change situation is purple (pH 7) → brown (pH 10) (Ran et al. 2025).

**TABLE 3:** Color response characteristics and applications of smart indicators based on CEO delivery system.

Indicator	Delivery system	Packaging form	Color variations	Food	References
Blackcurrant anthocyanins	Pickering emulsion	Starch/gelatin film	Taupe → khaki → dark green	Salmon	Lim et al. (2024)
Curcumin	Pickering emulsion	Gelatin/Chitosan Film	Yellow → orange → reddish brown	pork	Liu, Li, Chen, et al. (2022)
Ferulic acid co‐coloring of blueberry anthocyanins	Molecular inclusion	Potato starch‐based film	Bright red → blue‐purple	love bite	Li et al. (2023)
Roselle anthocyanins	nanofiber	PVDF film	Pink → Blue	pork	Zhang et al. (2022)
Oxidized mulberry extract	Pickering emulsion	Collagen Film	Red → black‐green	fishery	Ran et al. (2024)

During the spoilage process of meat, microorganisms decompose proteins to produce alkaline substances, which cause the pH value of the environment to rise. In the case of fruits, carbon dioxide is produced by respiration during storage, which lowers the pH value of the surrounding environment. Once the pH value is out of the range of the fresh state, the structure of anthocyanins changes, leading to a change in the color of the film. By observing the change in the color of the film, consumers can visually determine the freshness of the food. Rajendran et al. (2024) prepared a multifunctional composite film using anthocyanin‐rich onion skin extract and chitosan/CEO. The membrane simultaneously utilized the antimicrobial effect of CEO and the antioxidant synergistic effect of anthocyanins and was suitable for real‐time freshness monitoring of perishable foods such as meat (pH 5.5 → 7.5) and fish (pH 6.0 → 8.0). The amino group of chitosan binds to the phenolic hydroxyl group (‐OH) of anthocyanin through hydrogen bonding to stabilize the quinone structure and enhance the sensitivity of color development under alkaline conditions. The parent structure of onion skin anthocyanin (the main component is cyanidin‐3‐glucoside) contains 2‐phenylbenzopyran cation, and its color changes with pH through the reversible isomerization of oxonium salt (red) to quinone base (purple) to chalcone (yellow/brown).

This type of intelligent response system has two advantages over the traditional direct addition method: firstly, it releases the CEO only when the deterioration signal appears, avoiding the adverse effect of high concentrations of essential oils on food flavor; secondly, it dynamically adjusts the release rate according to the degree of deterioration, such as triggering the rapid release in high humidity or extreme pH environments, realizing the preservation of freshness through “on‐demand supply”. The second is to dynamically adjust the release rate according to the degree of deterioration, such as triggering rapid release in high humidity or extreme pH environments, to realize the “supply on demand” preservation strategy (Fernandes et al. 2022). By transforming the food deterioration signal into the driving force of material response, this technology not only exerts the antimicrobial activity of CEO but also avoids its defects, such as volatility and irritation, which provides a new paradigm for green and intelligent freshness preservation.

## 4 Industrialized Production

At present, the application of the CEO delivery system in food packaging is mostly in the laboratory research or small pilot stage; industrialized production faces many difficulties, specifically in the high cost of equipment, low production efficiency, expensive raw materials, and delivery system stability defects in four aspects (Fan et al. 2023). In order to promote the industrial production of the CEO delivery system, the key is to optimize the production process and explore alternative raw materials that are both inexpensive and have excellent performance. At the same time, new wall materials and embedding carriers should be actively explored to achieve a balance between large‐scale production and cost control.

From the production technology level, the high cost of precision equipment and the low efficiency of complex processes are the primary bottlenecks. The HPM of nanoemulsion and electrostatic spinning of nanofibers are not only a huge initial investment but also have high requirements of operation technology, which leads to the low production efficiency. In this regard, innovative preparation processes have become a breakthrough direction (Mohammadi et al. 2024). For example, multi‐spray nozzle array design can solve the problem of scale‐up efficiency of electrostatic spinning (Mohammadi et al. 2020). Spray drying–fluidized bed system can realize the integration of microcapsule encapsulation–drying and reduce energy consumption (Lim et al. 2021). Meanwhile, the process parameters of nanofiber electrospinning, Pickering emulsion stabilization, and microcapsule spray‐drying are optimized, and machine learning algorithms are combined to screen the optimal preparation conditions to reduce energy consumption and raw material loss (Klojdová and Stathopoulos 2022). Novel 3D printing technology can realize the personalization and rapid production of delivery systems. Chen, Zhang, et al. (2024) prepared gelatin‐polyvinyl alcohol‐carbon dots (GPC) outer layer + corn starch‐polyvinyl alcohol‐CEO (CPC) inner active bilayer membranes with an external barrier function and an internal controlled‐release effect using 3D printing technology. The banana peel carbon dots endowed the GPC membrane with strong antioxidant and UV‐blocking properties. Its interaction with the CPC membrane matrix is strongest when the CEO content is 3% (w/w). The bilayer structure significantly enhanced the stability and release of CEO, and the essential oil retention and release performance were optimal when the CPC layer was 60% filled. The membrane successfully retarded mango browning and rotting and extended the shelf life.

To overcome the technical challenges of poor stability and weak antimicrobial effect of single essential oils in food preservation applications, the antimicrobial properties of packaging materials can be improved by compounding essential oils. For example, Zeng et al. (2025) prepared microcapsules using spray‐drying technology, with HPCD as the wall material and a blend of CEO and clove essential oil as the core material. The optimal conditions for the preparation process were as follows: homogenization speed of 8000 r/min, wall material addition of 2%, HPCD to essential oil mass ratio of 1:3, essential oil‐to‐Tween‐80 mass ratio of 1.5:1, and homogenization time of 8 min. Experiments showed that the encapsulation rate of the prepared microcapsules was 65.82% ± 4.00%. This effectively inhibited the volatilization of essential oils and can be applied to the preservation of low‐salt pickled vegetables.

High production costs severely limit the industrial application of some carrier materials, so developing low‐cost, renewable alternative materials has become a research focus. In order to meet the environmental protection needs and reduce the pollution of packaging waste, the food industry can solve this problem through the strategies of waste biomass resource utilization and industrial by‐products to replace synthetic materials (Wei et al. 2024). For example, natural polymeric materials such as proteins or polysaccharides from sustainable sources such as plants are actively explored as encapsulation carriers. Rice husk cellulose and straw cellulose are less expensive to extract, and these materials are biodegradable, green, and widely available (Morimoto et al. 2023). Chitosan can also be prepared by deacetylation of crustacean processing waste (Liu, Li, Tang, et al. 2022). Microorganisms in industrial wastewater can be used to ferment and produce bacterial cellulose (Cesca et al. 2024). In addition, cellulose, pectin, and other polysaccharides can be extracted from agricultural by‐products such as hulls and straw (Almasi et al. 2020). Utilizing soy lecithin, a by‐product of the food industry, to replace synthetic lipids can significantly reduce the cost of liposome feedstock (Le et al. 2019). The development of green chemical synthesis pathways, such as the use of enzymatically modified chitosan or ultrasound‐assisted preparation of NEs, which reduces the use of organic solvents, can enhance the sustainability of the production process (Stefanowska et al. 2023). Chen, Feng, et al. (2025) prepared CEO‐loaded microcapsules (GE/PG‐CEOMs) by electrostatic compounding of gelatin (GE) and peach gum (PG). The encapsulation efficiency (EE) reached a maximum value of 90.8% when the core‐to‐wall ratio was 4:5, the wall concentration was 1.4%, and the pH was 3.8. The average particle size of the microcapsules was 50.9 μm. The microcapsules showed significant inhibition against *S. aureus* , *E. coli* , and Pseudomonas torulosa. The antimicrobial capsules containing GE/PG‐CEOMs were effective in delaying mushroom spoilage without direct contact.

In addition, stability issues are also industrialization bottlenecks, such as NEs that are susceptible to emulsion breakage due to temperature and pH changes, microcapsules that have wall deliquescence problems in high humidity environments, and some of the nanoparticles that have poor biodegradability (Song et al. 2024). Material composite technology can be used by the food industry to develop substrates with superior mechanical properties and environmental friendliness to improve the stability of the CEO delivery system. Vahedikia et al. (2019) prepared a biodegradable maize alcohol‐soluble protein film by doping 2% and 4% (w/w) of CEO with chitosan nanoparticles (CNPs), respectively. Experiments showed that the coated film could utilize the antimicrobial and film‐forming advantages of chitosan and improve the essential oil loading and slow‐release effect with the help of nanoparticles. The complexation of CEO with CNPs significantly enhanced the tensile strength of zeinolysin film but reduced its elongation at break. It was found that the addition of CEO alone or in combination with CNPs significantly inhibited the growth of *E. coli* and *S. aureus* , whereas the corn alkyd protein film loaded with CNPs alone did not have any significant inhibitory effect on the growth of microorganisms. Dag et al. (2023) developed a cellulose‐based active packaging film containing CEO. The film is based on hydroxypropyl methylcellulose (HPMC) and incorporates CEO through β‐CD encapsulation, cellulose nanocrystal Pickering emulsion (CNC‐P) emulsification, or a combination of both (CNC‐P/β‐CD). The film combines active freshness, heat sealability, and printability for high‐fat food packaging. Experiments showed that the addition of cellulose nanofibers enhanced the mechanical strength to 22.3 MPa, and the water vapor permeability of the CEO‐containing film remained stable at different humidity levels (33%–84% RH), with a significant increase in hydrophobicity. β‐CD and CNC‐P both delayed the release of CEO, and the antioxidant activity was maintained at more than 70%, which was superior to that of free CEO.

## 5 Discussion

As consumer demand for natural food additives and sustainable packaging continues to rise, research on CEO delivery systems is at a critical stage of transition from basic science to industrial applications. The industrialization of CEO delivery systems faces regulatory barriers at multiple levels, with regulatory differences in different regions significantly affecting the pathway to technology transfer (Jackson‐Davis et al. 2023). For example, in the United States, nanoscale delivery systems such as NEs or particles with particle sizes less than 100 nm are subject to additional safety assessments of the bioaccumulation and cytotoxicity of the nanomaterials (Motelica et al. 2023). EU regulations require active packaging materials to pass migration tests to ensure that the migration of cinnamaldehyde into food does not exceed the specified threshold. However, interface modification of nanocarriers may trigger additional safety assessment processes (Alonso et al. 2024; Bazilio et al. 2023). In addition, specific regulation of nanomaterials is a central barrier to industrialization. For example, the EU nanofood regulation requires food packaging containing nanoparticles to be clearly labeled as “nano,” while it remains controversial whether the particle size distribution of CEO NEs triggers this labeling (Istiqola and Syafiuddin 2020).

The industrialization of CEO delivery systems requires a balance between cost‐effectiveness and sensory compatibility, and encapsulation technologies can not only reduce odor interference but also enhance the functional properties of CEOs. For example, β‐cyclodextrin encapsulation can reduce the volatility of cinnamaldehyde through host‐guest interactions while enhancing its thermal stability. NEs need to strike a balance between surfactant dosage and odor masking (de Oliveira et al. 2024). For microencapsulation, the wall thickness should be optimized to avoid flavor fluctuations caused by “insufficient release at the beginning and sudden release at the end.” In the future, a dynamic balance between antimicrobial activity and flavor release can be achieved by screening high‐affinity wall materials through molecular docking technology or developing “odor‐responsive” carriers.

CEO delivery systems have shown potential for application in many fields with the help of NEs, microcapsules, liposomes, and other technologies. In the food industry, essential oils can be loaded onto composite membranes or coatings to inhibit Pseudomonas and *L. monocytogenes* and extend the shelf life of cold meat, fruits, vegetables, and dairy products (Sheerzad et al. 2024). In medicine, the nano delivery system can enhance the targeting of essential oils to effectively treat vaginal Candida infections, drug‐resistant bacterial infections, and oral inflammation (Ellboudy et al. 2023; Jeong et al. 2021; Lin et al. 2024). In agriculture, the CEO delivery system can repel rice pests, control fungal diseases in fruits and vegetables, and reduce pesticide use (Attia et al. 2023). In skin care, nanocarriers improve transdermal absorption of essential oils for acne treatment and anti‐aging (Xu et al. 2023). The core advantages of nanocarriers are targeted delivery, slow and controlled release, and good biocompatibility. However, toxicological evaluation and clinical translational research should be strengthened in the future to promote commercialization.

Sensory impact and label transparency are then key pain points for marketing. Nanoemulsion‐loaded CEOs may result in reduced light transmission in meat packaging films, affecting consumer judgment of food color (Khoder et al. 2025). pH‐responsive labels in smart packaging require consumers to understand the correlation between color and freshness. Although they can reflect freshness in real time, the logic of color change and freshness requires a certain level of consumer awareness.

In addition, existing studies have not paid enough attention to the long‐term toxicity of delivery materials and the migration pattern of active ingredients. When evaluating edible packaging styles, it is necessary to establish a “from molecule to human body” safety evaluation system. For example, the biocompatibility and intestinal mucosal permeability of nanoparticles should be evaluated in vitro by cell modeling, and the effects of long‐term intake of CEO delivery systems on liver and kidney function and intestinal flora should be studied in vivo by animal experiments (Paidari et al. 2021). At the same time, the migration rate of CEO and its carriers in different food matrices was systematically studied in accordance with the food contact material regulations of the European Union and the United States, and a risk assessment model was established to promote the development of standardized testing methods (Carvalho et al. 2022).

## 6 Conclusions

CEO delivery systems have shown significant potential for food preservation and active packaging applications due to their natural antimicrobial and antioxidant properties. Current research has effectively improved the volatility, water solubility, and odor interference of CEO through various technological means, such as microencapsulation, NEs, PEs, and molecular encapsulation. For example, NEs can enhance the antimicrobial efficiency of CEO and delay its release in food, while microencapsulation technology can achieve controlled release and stability of CEO through wall design. Intelligent packaging relies on the on‐demand release of CEO through pH or humidity response mechanisms, significantly extending the shelf life of perishable foods such as fruits, vegetables, and meat.

When choosing a suitable delivery system to encapsulate the CEO, the design principle, loading rate, slow release rate, performance, strengths and weaknesses, and stability of various delivery systems should be fully considered to maximize the industrial production benefits. However, industrial applications still face challenges such as high equipment costs for precision processes such as HPM and electrospinning, insufficient stability of nanocarriers, complex microencapsulation processes, and strict regulations on the safety assessment of nanomaterials. Future research should focus on the development of low‐cost wall materials, optimization of green preparation processes, and strengthening the research on interaction mechanisms between delivery systems and food matrices. Meanwhile, necessary evaluations should be conducted on food safety risks, and toxicological assessments and migration law analyses of delivery systems such as nanomaterials should be improved so as to promote the functional, intelligent, and large‐scale development of CEO delivery systems.

## Author Contributions

**Liquan Zhou:** conceptualization (equal), data curation (equal), formal analysis (equal), writing – original draft (lead). **Yahong Cai:** writing – review and editing (lead). **Yanjing Li:** formal analysis (equal), visualization (lead). **Yimiao Zhou:** conceptualization (equal), funding acquisition (equal), supervision (equal), writing – review and editing (equal). **Zuowei Xiao:** conceptualization (equal), funding acquisition (lead), supervision (equal), writing – review and editing (equal).

## Ethics Statement

The authors have nothing to report.

## Conflicts of Interest

The authors declare no conflicts of interest.

## Data Availability

The authors have nothing to report.
